# Genome composition and pollen viability of *Jatropha* (Euphorbiaceae) interspecific hybrids by Genomic *In Situ* Hybridization (GISH)

**DOI:** 10.1590/1678-4685-GMB-2019-0112

**Published:** 2020-01-31

**Authors:** Rosilda Cintra de Souza, Daniela de Argollo Marques, Marcel Mamede de Carvalho, Ana Rafaela da Silva Oliveira, Walter José Siqueira, Ana Maria Benko-Iseppon, Ana Christina Brasileiro-Vidal

**Affiliations:** 1 Universidade Federal de Pernambuco, Departamento de Genética, Recife, PE, Brazil.; 2 Universidade Federal Rural de Pernambuco, Departamento de Agronomia, Recife, Pernambuco, Brazil.; 3 Instituto Agronômico de Campinas, Campinas, São Paulo, Brazil.

**Keywords:** Interspecific crosses, Jatropha integerrima, J. multifida, physic nut, plant breeding

## Abstract

Interspecific hybridization is required for the development of *Jatropha curcas* L. improved cultivars, due to its narrow genetic basis. The present study aimed to analyze the parental genomic composition of F_1_ and BC_1_F_1_ generations derived from interspecific crosses (*J. curcas/J. integerrima* and *J. curcas/J. multifida*) by GISH (Genomic *In Situ* Hybridization), and the meiotic index and pollen viability of F_1_ hybrids. In F_1_ cells from both hybrids, 11 chromosomes of each parental was observed, as expected, but chromosome rearrangement events could be detected using rDNA chromosome markers, suggesting unbalanced cells. In the BC_1_F_1_, both hybrids had 22 chromosomes, suggesting that only *n* = 11 gametes were viable in the next generation. However, GISH allowed the identification of three and two alien chromosomes in *J. curcas//J. integerrima* and *J. curcas//J. multifida* BC_1_F_1_ hybrids, respectively, suggesting a preferential transmission of *J. curcas* chromosomes for both hybrids. Pollen viability in F_1_ hybrids derived from *J. curcas/J. integerrima* crosses were higher (82-83%) than those found for *J. curcas/J. multifida* (68%), showing post-meiotic problems in these last hybrids, with dyads, triads, polyads, and micronuclei as post-meiosis results. The here presented cytogenetic characterization of interspecific hybrids and their backcross progenies can contribute to the selection of the best genotypes for future assisted breeding of *J. curcas*.

## Introduction

The incorporation of renewable energy sources in the global energetic matrix is essential to ease the current and future energy crisis, considering the future shortage and the direct and indirect negative impact of petroleum and its derivatives to the environment, as pollutants. Known as physic nut, *Jatropha curcas* L. (Euphorbiaceae) has been considered one of the most promising oilseed plants for biodiesel and biokerosene production due to its productivity (yield ranges up to 3000 kg seeds/ha), high seed oil content and quality, reaching 40 to 50% (Sinha *et al.*, 2016), besides its ability to thrive in lands not suited to food crops (Carels, 2013; Montes and Melchinger, 2016). Despite the promises, *J. curcas* is an undomesticated species with no available stable and commercial cultivars that can make the energy culture feasible. Hence, there is a demand for continued investment in genetic breeding research (de Argollo Marques *et al.*, 2013). Additionally, *J. curcas* has been susceptible to numerous pests, such as white mite (*Polyphagotarsonemus latus*), bed bug (*Pachycoris torridus*), and green leafhopper (*Empoasca* spp.), besides several fungal diseases*.* Interspecific hybridization is a promising strategy for genetic enhancement of resistance of *J. curcas* against many biotic stresses (de Argollo Marques *et al.*, 2013; Sujatha, 2013).

The establishment, characterization and suitable use of a germplasm bank representing the genetic variability of the species (core collection) are essential for the success of a breeding program (Díaz *et al.*, 2017). Genetic diversity studies using morphological (Montes Osorio *et al.*, 2014; Pazeto *et al.*, 2015) or/and molecular markers (Basha and Sujatha, 2007; Tanya *et al.*, 2011; Sudheer *et al.*, 2011; Montes *et al.*, 2014; Pecina-Quintero *et al.*, 2014) have reported narrow diversity in *J. curcas* germplasm with exception of some studied Mexican accessions (probable center of origin of physic nut) (Santos *et al.*, 2016; Li *et al.*, 2017).

Generation of cultivars with higher productivity, increased oil content and quality, production uniformity, and resistance to biotic and abiotic stresses has been achieved by interspecific breeding (Sujatha, 2013). Congener species exhibit large genetic diversity, with several interesting agronomic traits (Popluechai *et al.*, 2009; de Argollo Marques *et al.*, 2013; Díaz *et al.*, 2017). The closely related evergreen shrub *J. integerrima* (Sudheer Pamidiamarri *et al.*, 2008) (2*n* = 2*x* = 22, Marinho *et al.*, 2018), for instance, carries traits not found in *J. curcas*, such as setting profuse flowers with uniform blooming on the same inflorescence, presence of woody stem and branches, besides dwarf varieties (Laosatit *et al.*, 2014, One *et al.*, 2014). Additionally, *J. integerrima* presents biotic stress tolerance, with maximum resistance against foliage feeders in terms of larval mortality, besides feeding cessation with or without pupation (Sujatha, 2013). On the other hand, the also diploid *J. multifida* (2*n* = 2*x* = 22, Marinho *et al.*, 2018) presents seeds about 30% larger and with a higher oil content (50%) than *J. curcas* (23-38%) (Sujatha, 1996; Banerji *et al.*, 1985). The energetic value of *J. multifida* oil (57.1 MJ/kg) is the highest among the studied *Jatropha* species, surpassing values observed for *J. glandulifera* Roxb. (47.2 MJ/kg), *J. gossypiifolia* L. (42.2 MJ/kg) and *J. curcas* (39.8 - 41.8 MJ/ kg) (Jones and Miller, 1991).

*Jatropha curcas* is a monoecious species with unisexual flowers (Montes and Melchinger, 2016); xenogamic (Divakara *et al.*, 2010; de Argollo Marques *et al.*, 2013); self-compatible (Chang-Wei *et al.*, 2007; Brasileiro *et al.*, 2012), and diploid (2*n* = 2*x* = 22), as well as most congeners species (Carvalho *et al.*, 2008; Sasikala and Paramathma, 2010; Marinho *et al.*, 2018). These traits allow crosses between *Jatropha* species, although with limited success due to either pre- or post-zygotic barriers (Moreira *et al.*, 2013), which can be overcome using *in vitro* embryo rescue technique (Laosatit *et al.*, 2017). However, several successful crosses have been reported, for instance, between *J. curcas* and *J. integerrima*, aiming at shorter plants, higher seed oil yield, resistance to diseases, woody biomass, etc (Sujatha and Prabakaran, 2003; Parthiban *et al.*, 2009; Muakrong *et al.*, 2014; One *et al.*, 2014).

Other difficulties may be related to problems in the meiotic and post-meiotic behavior of these hybrids, which can generate plants of little or no agronomic value, with low fertility or sterility due to reduction in the production of viable pollens and seeds. Thus, the evaluation of pollen viability is essential for the success of interspecific crosses (Pagliarini, 2000; Souza *et al.*, 2017), including *Jatropha* species. In addition, Genomic *In Situ* Hybridization (GISH) studies of interspecific hybrids can provide relevant information for breeding programs, allowing differentiation of the parental genomes in hybrid cells and the detection of non-homologous recombination, which is fundamental for the introgression of new traits into material derived from interspecific hybrids. GISH analyses may facilitate the choice of promising hybrids during the early stages of breeding through the detection of alien chromatin. This characterization allows the planning of crosses, aiming to maximize the segregation for the recovery of superior genotypes (Fukuhara *et al.*, 2016; Liu *et al.*, 2017; Ramzan *et al.*, 2017; Grewal *et al.*, 2018;).

Considering the limited knowledge regarding chromosome behavior, pollen viability and fertility of *Jatropha* interspecific hybrids and their progenies, the present work aimed to understand the chromosome behavior in F_1_ hybrids of *J. curcas*/*J. integerrima* and *J. curcas*/*J. multifida* and their respective BC_1_F_1_ backcrosses, inferring on their parental genomic composition, meiotic indexes and pollen viability. The presented results will facilitate the design of breeding programs for the improvement of wild trait introgressions to *J. curcas*.

## Material and Methods

### Plant material

*Jatropha curcas* and two congener species, *J. multifida,* and *J. integerrima* were used in interspecific crosses. Their F_1_ hybrids and backcrosses (BC_1_F_1_) were used to analyze the parental genomic composition by GISH, also evaluating post-meiotic behavior and pollen viability. Parents, crosses and respective accessions numbers are presented in Tables 1 and 2.

**Table 1:** *Jatropha curcas/J. integerrima* and *J. curcas/J. multifida* F_1_ hybrids, their backcrosses (BC_1_F_1_), and respective chromosome numbers.

Interspecific cross (Accessions^1^)	Generation	Accession*	Number of *J. curcas* chromosomes/Total (2*n*)
*J. curcas/J. integerrima* (L4P49/I2)	F_1_	L4V64	11/22
*J. curcas/J. integerrima* (L2P48/I5)		L3V50	11/22
*J. curcas/J. integerrima* (L4P37/I1)		L4V62	11/22
*J. curcas/J. multifida* (L13P43/M7)		L1V5	11/22
*J. curcas/J. multifida* (L12P35/M7)		L1V6	11/22
*J. curcas//J. curcas/J. integerrima* (L4P49//L4P49/I2)	BC_1_F_1_	L4V1	19/22
*J. curcas//J. curcas/J. multifida* (L3P18//R181)		L3VE	20/22

1 Accessions from Instituto Agronômico de Campinas (IAC).

**Table 2:** Pollen viability (%) of *Jatropha curcas/J. integerrima* and *J. curcas/J. multifida* F_1_ hybrid accessions based on the staining with Alexander reagent (1980).

Interspecific cross (Accessions^1^)	Accession (F_1_)^2^	Number of analyzed pollen grains	Number of viable pollen grains	Pollen viability (%)
*J. curcas/J. integerrima* (L2P34/I4)	L2V29	2500 **	2074	83%
*J. curcas/J. integerrima* (L5P3/I4)	L4V65	2500 **	2049	82%
*J. curcas/J. multifida* (L12P35/M7)	L1V6	2500 **	1700	68%

1 Accessions from the Instituto Agronômico de Campinas (IAC).

2 250 pollen grains analyzed per slide, with 10 slides per accession.

### F_1_ and BC_1_F_1_ hybrids

Hybridizations were performed according to Rulfino *et al.* (2013). Artificial pollination was carried out after emasculation and protection of developing female and male flowers. Elite *J. curcas* selected by the genetic breeding program of Instituto Agronômico de Campinas (IAC, Campinas, Brazil) was used as female parent in all crosses, while *J. multifida* and *J. integerrima* were used as male parents (for accession numbers see Tables 1 and 2). F_1_ seeds from these crosses were germinated on appropriate recipients until transference to field conditions. Afterward, during the F_1_ interspecific hybrids flowering, backcrosses were performed using *J. curcas* selected plants as recurring parental. For this step, we used female flowers of *J. curcas* and pollen of F_1_ hybrids.

### Mitotic chromosome preparation

For determination of parental genomic composition, root tips from both F_1_ or BC_1_F_1_ potted seedlings or plants were pre-treated with 2 mM 8-hydroxyquinolein (8-HQ) for 4.5 h at 18 °C, fixed in methanol:acetic acid (3:1, v/v) for at least 4 h and then stored at -20 °C. Next, they were washed three times in distilled water and digested in a 2% cellulase (w/v, Onozuka R-10, Serva) and 20% pectinase (v/v, Sigma-Aldrich) solution for 4 h at 37°C.

Slide preparation followed Carvalho and Saraiva (1993) with modifications introduced by Vasconcelos *et al.* (2010). Best slides were selected for staining in 4’,6-diamidino-2-phenylindole (DAPI) (2 μg/mL):glycerol (1:1, v/v). Subsequently, they were destained in ethanol:glacial acetic acid (3:1, v/v) for 30 min and transferred to absolute ethanol for 1 h, both at room temperature. After air-dried, the selected slides were stored at -20 °C until GISH and FISH experiments were performed.

### DNA probes and labeling

For FISH procedures, the following probes were used: (1) R2, a 6.5 kb fragment containing the 18S-5.8S-25S rDNA repeat unit from *Arabidopsis thaliana* (L.) Heynh. (Wanzenböck *et al.*, 1997), and (2) D2, a 400 bp fragment containing two 5S rDNA repeat units from *Lotus corniculatus* L. [as *L. japonicus* (Regel) K.Larsen] (Pedrosa *et al.*, 2002), which were labeled by nick translation with digoxigenin-11-dUTP (Roche Diagnostics) and biotin-11dUTP (Sigma), respectively.

For GISH analyses, genomic DNA was extracted according to Weising *et al.* (2005) and resuspended in Milli-Q water. Subsequently, DNA samples were treated with RNAse and quantified in 1% agarose gel. For probe labeling, genomic DNA samples of *J. integerrima* and *J. multifida* were labeled with digoxigenin-11-dUTP (Roche) by nick translation (Roche Diagnostics, Life Technologies). For blocking, non-labeled genomic DNA of *J. curcas* was fragmented (200-500 bp) by autoclaving.[Bibr B55]

### Fluorescent *In Situ* Hybridization (FISH) and Genomic *In Situ* Hybridization (GISH)

Pre-treatments and post-hybridization washes were based on Pedrosa-Harand *et al.* (2009), in which the stringency wash was performed with 0.1 saline-sodium citrate (SSC) at 42 °C. Chromosome and probe denaturation and detection were performed according to Heslop-Harrison *et al.* (1991). The hybridization mixture, containing 50% formamide (v/v), 2 SSC, 10% dextran sulfate (w/v) and 5 ng/μL of the probe, was denatured at 75 °C for 10 min. For the GISH preparations, *J. curcas* blocking DNA was also added to the hybridization mixture. Different probe:blocking ratios were tested for both hybrids (1:0 to 1:40 for *J. integerrima*:*J. curcas*, and 1:10 to 1:60 for *J. multifida*:*J. curcas*). For hybrid analyses, ratios of 1:40 and 1:60 were used for *J. integerrima*:*J. curcas* and *J. multifida*:*J. curcas*, respectively. Slides were denatured at 85 °C for 7 min. After GISH procedures, reprobing of slides for localization of 5S and 35S rDNA in the same cell was performed up according to Heslop-Harrison *et al.* (1992).

Digoxigenin-labelled probes were detected using sheep anti-digoxigenin-FITC (Roche Diagnostics) and amplified with donkey anti-sheep-FITC (Sigma), in 1% (w/v) BSA. Biotin-labelled probes were detected with mouse anti-biotin (Dako), and the signal was visualized with rabbit anti-mouse TRITC conjugate (Dako), in 1% (w/v) BSA. All preparations were counter-stained and mounted with 2 μg/mL DAPI in Vector’s Vectashield (1:1; v/v).

Images of the best cells were acquired using a Leica DMLB epifluorescence microscope and a Leica DFC 340FX camera with the Leica CW4000 software. Images were pseudocolored and optimized for contrast and brightness with Adobe Photoshop CS4 (Adobe Systems Incorporated) software.

### Post-meiotic assays

For post-meiotic analyses, flower buds were fixed in ethanol:glacial acetic acid (3:1, v/v), for 6 h at room temperature and stored at -20 °C. Subsequently, anthers were digested in 2% (w/v) cellulase Onozuka R-10 (Serva), 1% (w/v) pectolyase (Sigma-Aldrich), and 1% (w/v) cytohelicase (Sigma-Aldrich) for 4 h at 37 °C. Then, they were washed in distilled water, squashed and stained in 2% acetic carmine. Five slides were analyzed per hybrid. The quantities of the post-meiotic products (dyads, triads, tetrads, and polyads) were registered for the calculation of meiotic index, by dividing the normal tetrad number by the total post-meiotic products multiplied by 100. Tetrads with four cells exhibiting uniform size were considered as normal post-meiotic products. On the other hand, dyads, triads, and polyads were considered abnormal.

For determination of pollen viability, flower buds in pre-anthesis were fixed in ethanol:glacial acetic acid (3:1, v/v), for 6 h at room temperature and stored at -20 °C. Subsequently, anthers were transversally sectioned, and pollen grains were released in Alexander solution (Alexander, 1980) for staining and observation by light microscopy. Through this test, viable pollen presents the purple color in protoplasts and green in the cellulose wall, while non-viable grains stain only in green or blue. Ten slides were analyzed per hybrid: *J. curcas/J. integerrima* (F_1_: L2V29, L4V65) and *J. curcas/J. multifida* (F_1_: L1V6), with 250 pollen grain per slide totalizing 2,500 pollen units per hybrid.

## Results

### GISH and FISH in mitotic chromosomes

The F_1_ hybrids between *J. curcas/J. integerrima* and *J. curcas/J. multifida* presented no chromosomal loss in mitosis, maintaining the diploid chromosome number (2*n* = 22).

**Figure 1:**
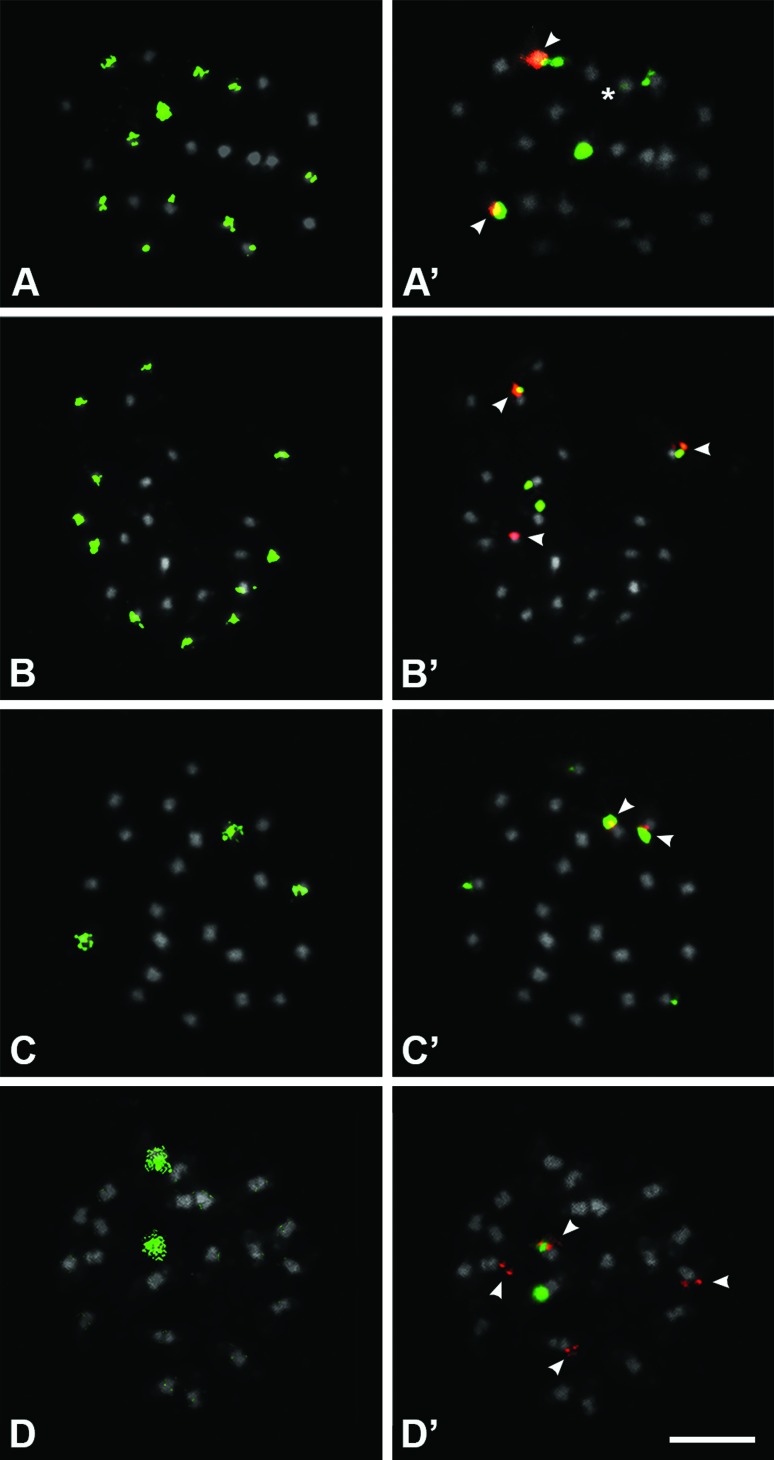
Genomic *In Situ* Hybridization (GISH, **A**-**D**) and Fluorescent *In Situ* Hybridization (FISH, **A’**-**D’**) in mitotic metaphases of hybrids between *J. curcas* and *J. integerrima* (**A**, **C**) and between *J. curcas* and *J. multifida* (**B**, **D**) generation F_1_ (**A**, **B**) and BC_1_F_1_ (**C**, **D**). DAPI counterstained chromosomes (pseudocolored in gray), genomic probes (in green) of *J. integerrima* (**A**, **C**) and *J. multifida* (**B**, **D**). (**A**, **B**) F_1_ hybrids with 11 chromosomes from each parental, being those not marked of *J. curcas*. (**C**) *J. curcas*//*J. curcas*/*J. integerrima* BC_1_F_1_, evidencing three *J. integerrima* chromosomes in green. (**D**) *J. curcas*//*J. curcas*/*J. multifida* BC_1_F_1_, showing two *J. multifida* chromosomes in green. 5S rDNA (pseudocolored in red and indicated by arrowheads) and 35 rDNA (pseudocolored in green) (**A’**-**D’**). Asterisk in **A’** indicate a faint rDNA site. Bar in **D’** represents 5 μm.

Application of GISH to interspecific *Jatropha* hybrids was possible in mitotic metaphases, although their cells have small, morphologically similar chromosomes. However, for some chromosomes, especially the less condensed prometaphase ones, hybridization of the late condensing subterminal regions was not possible. In such cases, the signals were restricted to terminal dots and the heterochromatic pericentromeric region (Figure 1A, B).

In regard to the three *J. curcas/J. integerrima* F_1_ hybrids (L4V64, L3V50, and L4V62), the GISH evidenced that half of the chromosome set (11 chromosomes) was originated from *J. integerrima*, whereas the remaining 11 *J. curcas* chromosomes remained unmarked, as expected for this generation (Figure 1A). From *J. curcas* chromosomes, one presented adjacent 5S and 35S rDNA sites (being the 35S rDNA more distal), and two chromosomes had only terminal 35S rDNA, but different in size. From *J. integerrima* chromosomes, one had both 5S and 35S rDNA sites and another presented a smaller faint 35S rDNA (Figure 1A’).

Likewise, the cells of the two hybrids of *J. curcas/J. multifida* of the F_1_ generation (L1V5 and L1V6) presented 11 chromosomes hybridized with *J. multifida* probe and 11 unmarked *J. curcas* chromosomes (Figure 1B). From *J. curcas* chromosomes, one presented adjacent 5S and 35S rDNA sites, two had only terminal 35S rDNA, and one had only one terminal 5S rDNA. From *J. multifida* chromosomes, only one had both 5S and 35S rDNA sites (Figure 1B’).

In the cells of the *J. curcas//J. curcas/J. integerrima* hybrid, in generation BC_1_F_1_ (L4V1), the probe of *J. integerrima* hybridized to only three out of 22 chromosomes (Figure 1C), demonstrating that most chromosomes originated from *J. curcas*. From *J. curcas* chromosomes, one presented adjacent 5S and 35S rDNA sites (being the 35S rDNA more distal), and three had only small terminal 35S rDNA with different sizes. From *J. integerrima* chromosomes, only one had both 5S and 35S rDNA sites (Figure 1C’).

Similarly, the *J. multifida* probe hybridized in only two of the 22 chromosomes of *J. curcas//J.curcas/J. multifida* hybrid in generation BC_1_F_1_ (L3VE), thus evidencing that the other 20 chromosomes originated from *J. curcas* (Figure 1D). From *J. curcas* chromosomes, one presented only terminal 35S rDNA site, and three had only small terminal 5S rDNA site. From *J. multifida* chromosomes, only one had both 5S and 35S rDNA sites (Figure 1D’).

### Post-meiotic assays

**Figure 2:**
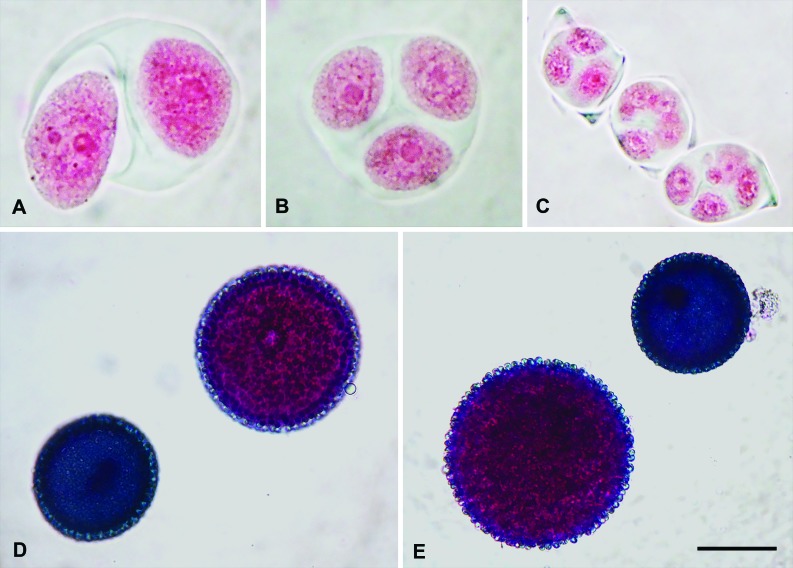
Pollen viability and post-meiotic stage (tetrad formation) analysis in *Jatropha* hybrids and related species in the F_1_ generation, L1V6 accession (*J. curcas*/*J. multifida*) (**A, B, D**), L2V29 (*J. curcas*/*J. integerrima*) (**C, E**). (**A, B, C**) Post-meiotic phases stained with 2% Carmine acetic with formation of (**A**) dyad, (**B**) triads and (**C**) unbalanced tetrads with nuclei of distinct sizes. (**D, E**) Pollens stained with reactive of Alexander (1980), in pink, viable pollen and, in blue, infeasible pollen. Bar in **E** represents 5 μm.

In the anthers of L2V29 and L4V65 (F_1_ hybrids; *J. curcas/J. integerrima*), a predominance of normal tetrads (90% and 85%, respectively) was observed, although some tetrads with micronuclei were visualized in about 10 and 15% of the material analyzed, respectively (Figure 2). On the other hand, the L1V6 (F_1_ hybrid; *J. curcas/J. multifida*) presented abnormal post-meiotic products, including tetrads with micronuclei, dyads, triads or polyads in 90% of the analyzed material (Figure 2A-C).

The pollen viability of both F_1_ hybrids of *J. curcas*/ *J. integerrima* (L2V29 and L4V65) varied from 82 to 83% (Table 2, Figure 2D-E), while viability was reduced to 68% in the F_1_ hybrid of *J. curcas*/*J. multifida* (L1V6).

## Discussion

Although there is previous work on the parental genomic composition of *J. curcas* and *J. integerrima* interspecific hybrids (Fukuhara *et al.*, 2016), this is the first cytogenetic study analyzing hybrids of F_1_ and BC_1_F_1_ generations derived from a cross between *J. curcas* and *J. multifida*. Besides, the present work regards the second evaluation of parental genomic composition, post-meiotic behavior and pollen viability in *Jatropha* interspecific hybrids. Adjusted GISH methodology for *Jatropha* species enabled to increase efficiency in obtaining improved cultivars through interspecific crosses and assisted selection. Although *Jatropha* species have small chromosomes, the GISH technique allowed the distinction of chromosomes of both parental genomes in the here evaluated interspecific hybrids.

However, GISH pattern for *Jatropha* chromosomes appears mainly at pericentromeric region, probably due to their heterochromatic proximal condensation pattern (Fukuhara *et al.*, 2016), in accordance to the CMA^+^ (Chromomycin A3) heterochromatin distribution for *J. curcas* chromosomes, for instance (Marinho *et al.*, 2018), indicating the preferential GISH for heterochromatic regions. The pericentromeric heterochromatin in *J. curcas* is constituted in part by *Gypsy-*type retrotransposon (Alipour *et al.*, 2014). On the other hand, in *J. integerrima* and in *J. multifida* species, the heterochromatic CMA^+^ pattern is restricted to 35S rDNA sites (Marinho *et al.*, 2018). Additionally, terminal dots in *J. curcas* probably correspond to JcSat1 *J. curcas* satellite DNA sequence (Fukuhara *et al.*, 2016) or to *Copia-*type elements as described previously (Alipour *et al.*, 2013). However, no terminal heterochromatic dots were found for *J. integerrima* or *J. multifida* chromosomes (Marinho *et al.*, 2018), as was corroborated by the absence of dot sites by GISH for chromosomes of both species in F_1_ hybrids in the present work and by Fukuhara *et al.* (2016) for *J. curcas* x *J. integerrima* hybrids. Previous work with GISH on species with small chromosomes also showed preferential *in situ* hybridization in regions rich in repetitive DNA, as observed in *Arachis* (Seijo *et al.*, 2007) and *Cucumis* (Zhang *et al.*, 2015).

Regardless of the pollen donor species (*J. integerrima* or *J. multifida*), the here evaluated F_1_ hybrids showed the expected chromosome number in mitotic metaphases (11 chromosomes originating from each parental), resulting in a normal diploid number (2*n* = 22) in all analyzed cells. Additionally, for both F_1_ hybrids, one carrier 5S and 35S rDNA chromosome were identified per genome as expected (see Marinho *et al.*, 2018). However, for *J. curcas/J. integerrima* two 35 rDNA carrier chromosomes were observed for the *J. curcas* chromosomes and one chromosome with a faint site was observed for the *J. integerrima* instead of one per genome as expected (see Marinho *et al.*, 2018). Also, for *J. curcas/J. multifida*, both 35 rDNA carrier chromosomes were from *J. curcas*. These data indicate that, despite the apparently mitotic stability for both F_1_ hybrids, there was an apparently preferential presence of the 35 rDNA carrier *J. curcas* chromosomes for both F_1_ hybrids. Additionally, the extra 35 rDNA faint site for *J. curcas/J. integerrima* and the extra 5 rDNA site for *J. curcas/J. multifida* indicate chromosome rearrangement events for both hybrids and possible unbalanced chromosomes.

Previous data for the hybrids analyzed revealed only 21.1% fruit yield rate for the crosses between *J. curcas* and *J. integerrima*, indicating a high abortion rate, although the germination of the hybrid in question was high (83.5%) (Rulfino *et al.*, 2013). Regarding *J. multifida*, the observed incompatibility was still higher; of the 582 crosses conducted, only 45 fruits were produced (7.6% success), and only four seeds germinated. According to Moreira *et al.* (2013), the low fruit index derived from these crosses resulted in post-zygotic genetic incompatibility.

Morphological features observed in both F_1_ interspecific hybrids were intermediate between female (*J. curcas*) and male (*J. integerrima* or *J. multifida*) parental species (Rulfino *et al.*, 2013), corroborating GISH results related to parental chromosome distribution (i.e., 11 chromosomes for each parental). The morphological variability observed in F_1_ population was high for both interspecific hybridization assays. For instance, *J. curcas/J. integerrima* population presented plants with variation in size (dwarf, semi-dwarf, medium and high), leaf pigmentation and shape (anthocyanin), flower coloration (light pink to purple), size and number of female and male flowers (Figure S1). Similarly, the morphological traits of *J. curcas*/*J. multifida* plants were also intermediate showing, e.g., seven leaf lobes in the F_1_ hybrid which is intermediate of *J. curcas* (five) and *J. multifida* (nine) (Figure S2). Flower colors of these interspecific hybrids were also different from the male and female parental (Figures S1, S2).

On the other hand, concerning reproductive structures, all F_1_ hybrids showed similarity with the male parental (*J. multifida* or *J. integerrima*) (Rulfino *et al.*, 2013). Pollen viability of *J. curcas/J. integerrima* F_1_ hybrids was high (82 to 83%) in the present work, higher than previous reported for *J. curcas* (77%) and *J. integerrima* (72.5%) species (Rufino *et al.*, 2013). It allowed the advancement of generations, with a high rate of seed formation in BC_1_F_1_ generation (*J. curcas*//*J. curcas*/*J. integerrima*), with 31% pollen-fruit setting and 85.9% seed germination, due to higher genetic compatibility between *J. curcas* and plants of the F_1_ generation. However, the post-germination survival rate was low (38.9%) (Rufino *et al.*, 2013), probably due to the expression of damaging alleles in BC_1_F_1_ plants. In contrast, low rates were found for these hybrids in previous studies, with respect to pollen viability of F_1_ hybrids (average rate of 48.4%), probably associated to several meiotic abnormalities observed (Fukuhara *et al.*, 2016), which presented low frequency in the present work (10-15%). The same situation applies to the seed setting in F_2_ hybrids of *J. curcas/J. integerrima* (Sujatha and Prabakaran, 2003; Parthiban *et al.*, 2009; Muakrong *et al.*, 2014), as compared with the present results. Such divergent results may be related to the different genotypes used in the crosses of both works or, still, to environmental factors. According to Müller *et al.* (2016), sexual reproduction is very sensitive to environmental perturbations, and pollen viability can vary accordingly under high temperature and in thermotolerant genotypes, as observed for cultivated tomato (*Solanum lycopersicum*).

In turn, the pollen viability observed in the F_1_ hybrid of *J. curcas/J. multifida* was relatively low (68%), but similar to observed previously for *J. multifida* species (68%) (Rulfino *et al.*, 2013). This reduction in viability may be directly associated with the observed meiotic irregularities of this hybrid (L1V6, F_1_ *J. curcas/J. multifida*), especially considering post-meiotic irregularities, such as the formation of dyads, triads, polyads and micronuclei, compromising the pollen viability and possibly leading to a reduction of vigor and fertility (Fuzinatto *et al.*, 2008; Reis *et al.*, 2008). According to Rulfino *et al.* (2013), the male flowers of the F_1_ hybrid resulting from the crossing with *J. multifida* generated smaller pollen grains, not visible to the naked eye. Despite this, these plants were used to pollinate female flowers of *J. curcas*, allowing the production of the BC_1_F_1_, but with low of fruit setting (7.6%) and seed germination (8.8%) rates (Rulfino *et al.*, 2013). The resulting meiosis behavior observed in the present work may explain the inferior performance of this interspecific cross and is in accordance to their phylogenetic distance (Sudheer Pamidiamarri *et al.*, 2008).

Both BC_1_F_1_ interspecific hybrids here evaluated (*J. curcas*//*J. curcas*/*J. integerrima* and *J. curcas*//*J. curcas*/*J. multifida*) exhibited 22 chromosomes in all analyzed mitotic metaphases, suggesting that only *n* = 11 gametes were feasible for the formation of the new generation, although the formation of aneuploid microspores for *J. curcas/J. integerrima* F_1_ hybrids was reported by Fukuhara *et al.* (2016). In the present work, a preferential presence of *J. curcas* chromosomes for both BC_1_F_1_ hybrids was observed, as previously reported to S_1_ individuals obtained by self-pollination of *J. curcas/J. integerrima* F_1_ (Fukuhara *et al.*, 2016). Only two or three alien chromosomes were observed in BC_1_F_1_ plants, differing from the expected number (11 + 5 or 6 from *J. curcas* and 5 or 6 from related species), probably because this species was used as a recurrent female parent, both in the present work and in Fukuhara *et al.* (2016). However, we cannot infer if the preferential transmission was affected by cytoplasmic factors, because no reciprocal crosses were performed in the present work. Additionally, for *J. curcas*//*J. curcas*/*J. multifida*, the three extra 5S rDNA sites in separate chromosomes, besides single carrier 5S-35S rDNA and 35S rDNA chromosomes indicate chromosome rearrangements.

A higher number of *J. curcas* chromosomes in BC_1_F_1_ resulted in plants with more similar phenotypes to this parental species. In *J. curcas*/ *J. integerrima* hybrids (including those studied in this work, L4P49 e L3P18), for example, most of the BC_1_F_1_ hybrids (90%) presented leaves with the characteristic pentagonal form (“curcas type”), whereas in few individuals the leaves were lanceolate (Figure S3), similar to *J. integerrima* (Rulfino *et al.*, 2013). This similarity with *J. curcas* seems to reflect the loss of most *J. integerrima* chromosomes in this generation. Other features deserve mentioning, such as flower and seed color, fruit shape, number of female flowers, number of fruits per bunch, number of bunches per plant, resistance to pests and diseases, oil content and quality, phorbol esters contents, which were quite variable among plants of the BC_1_F_1_ generation (unpublished data). The lower size (dwarf) characteristic of *J. integerrima* male parental and erect growth (characteristic of the female parental *J. curcas*) also segregated in the BC_1_F_1_ population (Rulfino *et al.*, 2013). However, most of the obtained hybrids had phenotypic characteristics closer to the female parental (*J. curcas*)*.*

Similarly, the few and unpublished BC_1_F_1_ hybrids generated from the cross between *J. curcas* and *J. multifida* showed greater resemblance with the recurrent parental *J. curcas*, although characteristics as fruit shape, seed and oil yield, phorbol esters content presented interesting variability (progenies under investigation). It should be noted that most of the hybrids obtained, including those studied in the present work (Table 1), exhibited phenotypic characteristics closer to the female parental *J. curcas* than that of the parent pollen donors (*J. integerrima* or *J. multifida*), corroborating the higher number of chromosomes of *J. curcas* observed after GISH analyzes.

Continuous selection of plants with characteristics of interest among the backcrossing hybrids (BC_1_F_1_) may result in plants with agronomical interesting features in medium to long term. For instance, the selection of lower size plants with introduced (alien) chromosomes of *J. integerrima* can be promising for the production of viable cultivars to the mechanized harvest, with consequent reduction of production costs. In this sense, GISH can help in the future characterization and selection of the best genotypes, aiming at the advance and planning of the next crosses towards a stable *J. curcas* cultivar.

Despite the meiotic abnormalities found in the F_1_ generation and the reduction of pollen viability, especially for the crossing of *J. curcas/J. multifida*, the number of regular pollen grains was sufficient to allow generation advance (BC_1_F_1_) with hybrids bearing a stable chromosomal number (2*n* = 22) equal to the parental individuals for all analyzed mitotic metaphases. This indicates that only gametes with *n* = 11 chromosomes were feasible for the formation of the new generation, but chromosome rearrangement events could be detected using rDNA chromosome markers, suggesting unbalanced cells. On the other hand, GISH results uncovered that BC_1_F_1_ hybrid individuals presented a higher number of chromosomes from *J. curcas* recurrent parental than expected, indicating a preferential transmission of chromosomes from this species.

## Supplementary material

The following online material is available for this article:

Figure S1 F1 interspecific hybrid of the cross between *J. curcas (♀ )* and *J. integerrima (♂ )*.

Figure S2 Interspecific cross between *J. curcas (♀ )* and *J. multifida (♂ )*.

Figure S3 BC1F1 Interspecific hybrid of cross between F1 (*J. curcas/J. integerrima*) and *J. curcas*.
