# Supplementary material for: Genome composition and pollen viability of Jatropha (Euphorbiaceae) interspecific hybrids by Genomic In Situ Hybridization (GISH)
Source: Genet Mol Biol. 2020 Jan 31;42(4):e20190112. doi: 10.1590/1678-4685-GMB-2019-0112 (PMC7198012; doi:10.1590/1678-4685-GMB-2019-0112)
Supplement: Supplementary file 1 [file 1415-4757-GMB-42-4-e20190112-s1.pdf]

Supplementary Material to "Genome composition and pollen viability of *Jatropha* (Euphorbiaceae) interspecific hybrids by Genomic *In Situ* Hybridization (GISH)"

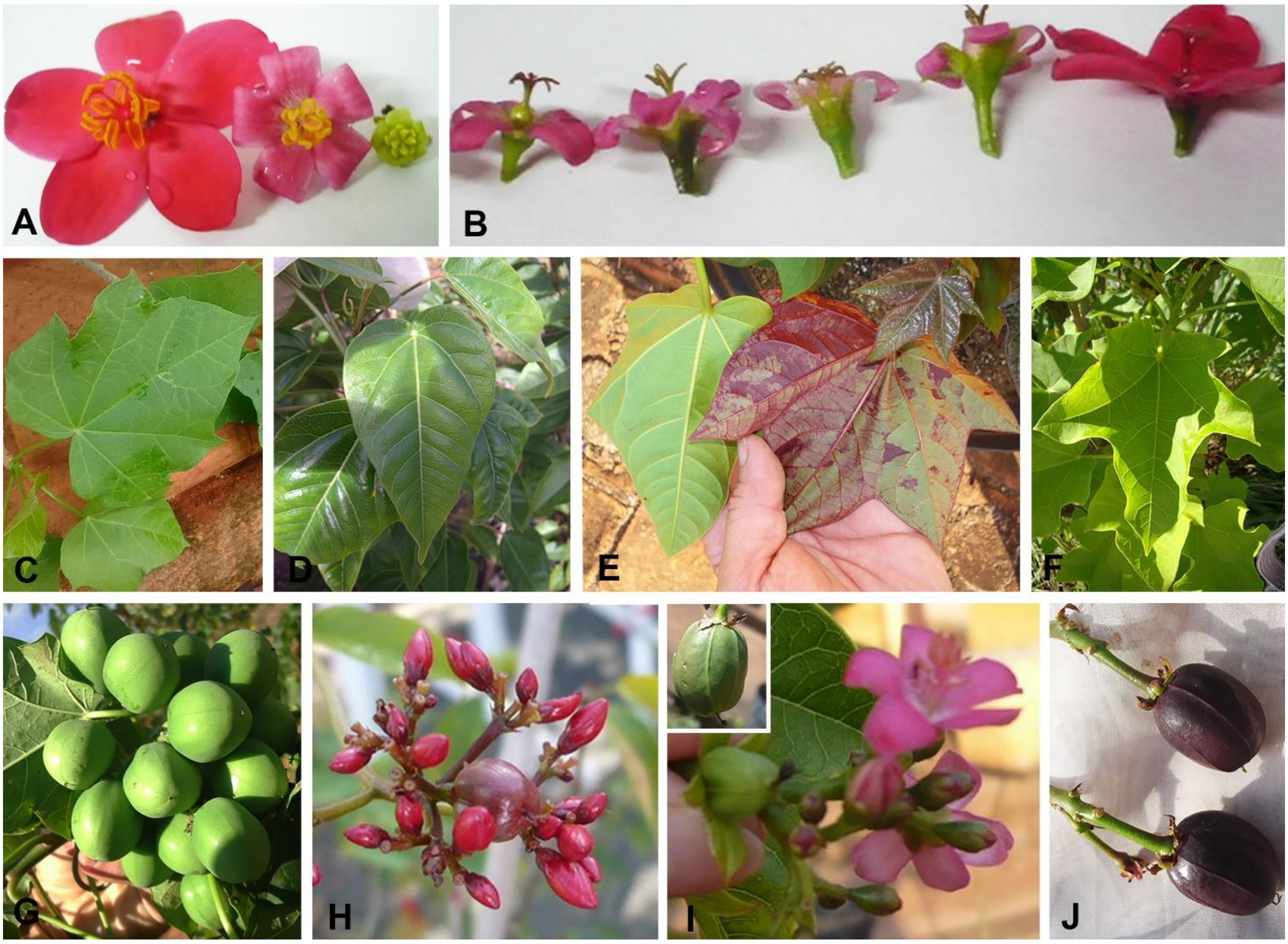

Figure S1 - F_1 interspecific hybrid of the cross between *J. curcas* (♀) and *J. integerrima* (♂). Flower color, size and shape variations in F_1 plants: (**A**) pink F_1 hybrid male flower between red *J. integerrima* (left) and green *J. curcas* (right); (**B**) female flower segregation on F_1 hybrid. Leaf shape and pigmentation segregation: (**C**) *J. curcas*, (**D**) *J. integerrima*, and (**E, F**) F_1 hybrid plants. Fruit shape and color segregation: (**G**) *J. curcas*; (**H**) *J. integerrima*; (**I**) green (insert) and (**J**) purple colored fruit in F_1 plants.
