# Supplementary material for: Genome composition and pollen viability of Jatropha (Euphorbiaceae) interspecific hybrids by Genomic In Situ Hybridization (GISH)
Source: Genet Mol Biol. 2020 Jan 31;42(4):e20190112. doi: 10.1590/1678-4685-GMB-2019-0112 (PMC7198012; doi:10.1590/1678-4685-GMB-2019-0112)
Supplement: Supplementary file 2 [file 1415-4757-GMB-42-4-e20190112-s2.pdf]

Supplementary Material to "Genome composition and pollen viability of *Jatropha* (Euphorbiaceae) interspecific hybrids by Genomic *In Situ* Hybridization (GISH)"

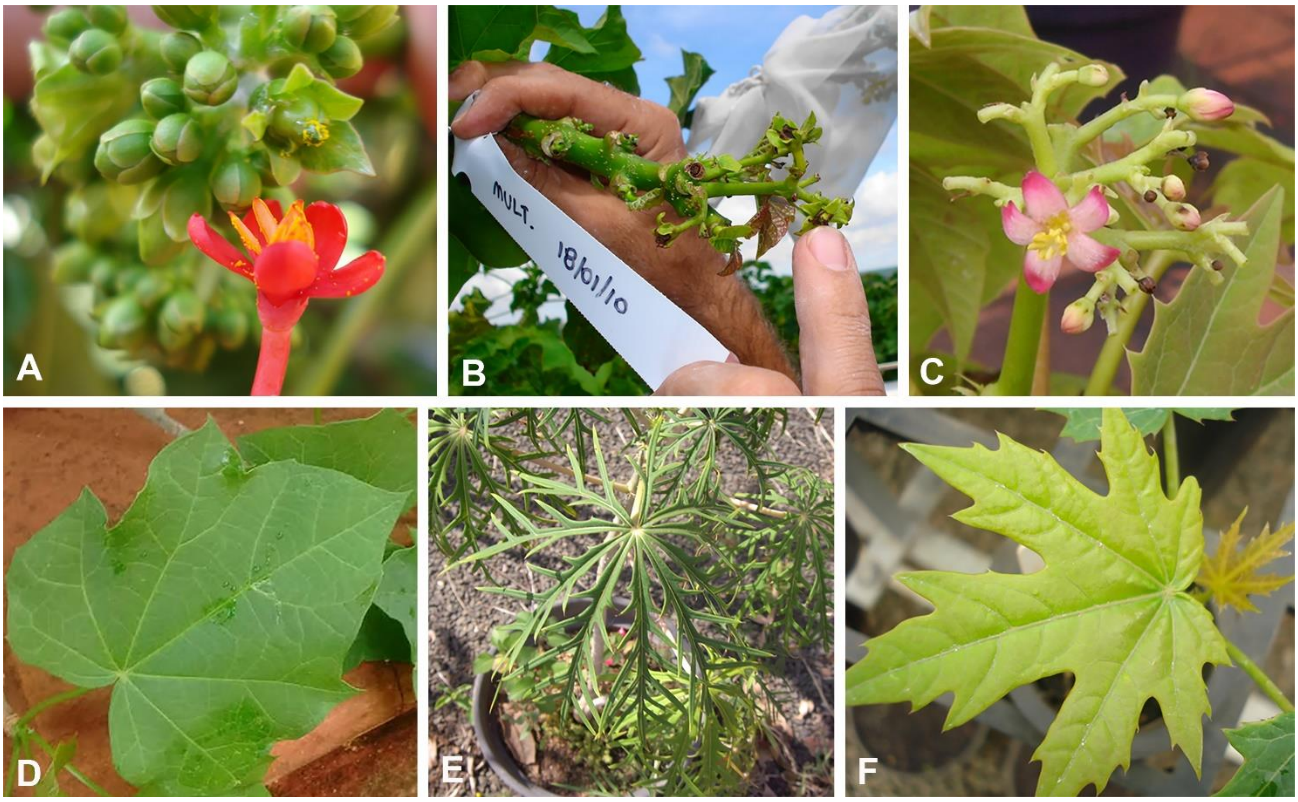

Figure S2 - Interspecific cross between *J. curcas* (♀) and *J. multifida* (♂) showing: (**A**) red flower of pollen donor *J. multifida* and green flower of *J. curcas;* (**B**) fruit fixation: aborted and non-aborted fruits (finger pointed); (**C**) intermediate color of F_1 hybrid flower. Leaf shape: (**D**) *J. curcas* with five leaf lobes (five); (**E**) *J. multifida* with nine, and (**F**) F_1 hybrid with intermediate shape with seven lobes.
