# Supplementary material for: Genome composition and pollen viability of Jatropha (Euphorbiaceae) interspecific hybrids by Genomic In Situ Hybridization (GISH)
Source: Genet Mol Biol. 2020 Jan 31;42(4):e20190112. doi: 10.1590/1678-4685-GMB-2019-0112 (PMC7198012; doi:10.1590/1678-4685-GMB-2019-0112)
Supplement: Supplementary file 3 [file 1415-4757-GMB-42-4-e20190112-s3.pdf]

Supplementary Material to “Genome composition and pollen viability of *Jatropha* (Euphorbiaceae) interspecific hybrids by Genomic *In Situ* Hybridization (GISH)”

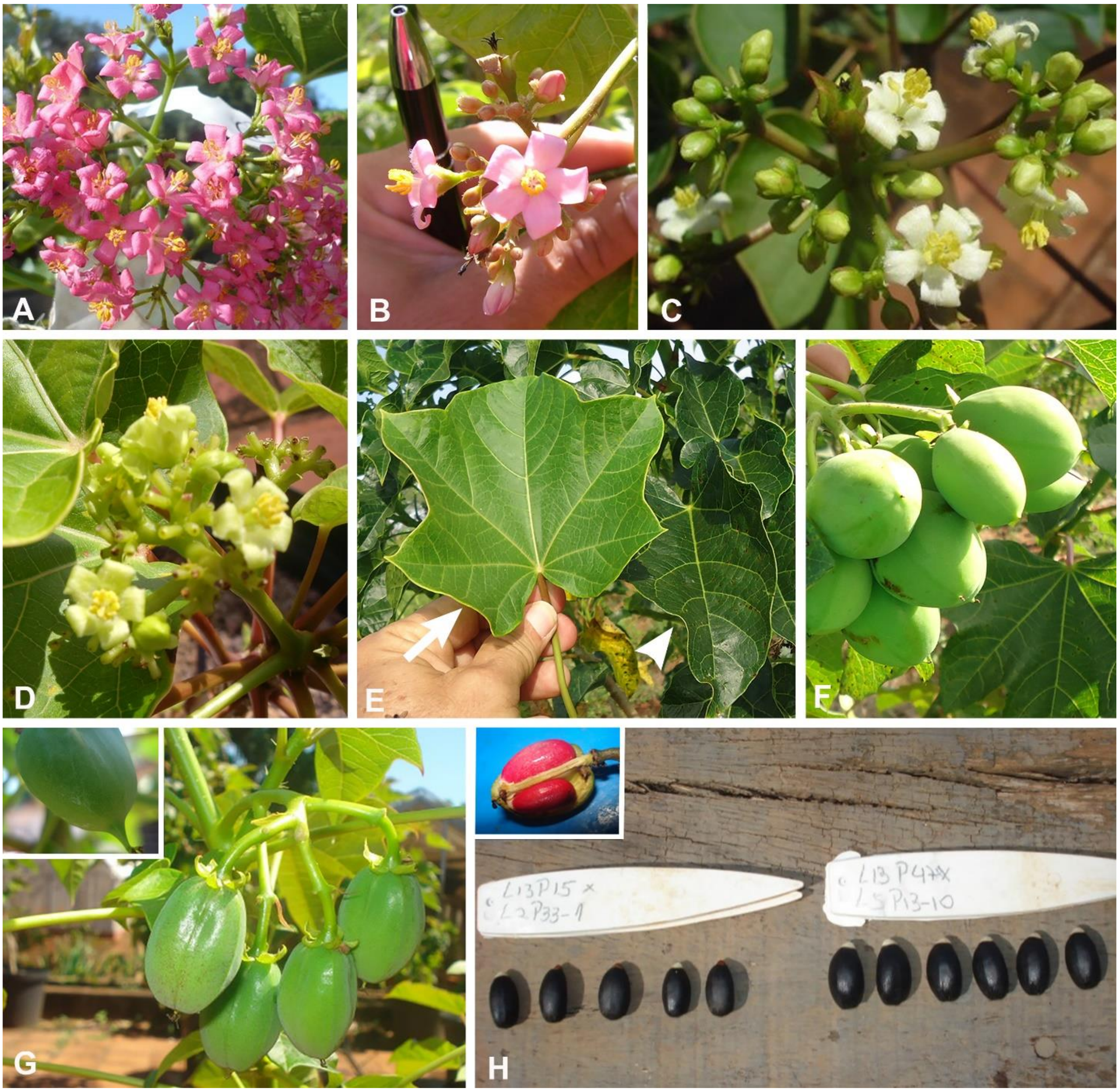

Figure S3 - BC_1F_1 Interspecific hybrid of cross between F_1 (*J. curcas*/*J. integerrima*) and *J. curcas*, showing morphological variation on flowers with regard to: (**A, B, C, D**) number and color of flowers; (**E**) leaf format variation: lanceolate leaf similar to the male parental *J. integerrima* (arrowhead) and a pentagonal leaf typical of the female parental *J. curcas* (arrow). (**F, G**) Fruit variation, and (**H**) seed size variation.
